# Experience of Dry Eye Patients With Anxiety and Depression: A Qualitative Study

**DOI:** 10.3389/fmed.2022.830986

**Published:** 2022-03-31

**Authors:** Haozhe Yu, Weizhen Zeng, Minhui Xu, Wenyu Wu, Yun Feng

**Affiliations:** Department of Ophthalmology, Peking University Third Hospital, Beijing, China

**Keywords:** dry eye disease, anxiety, depression, coping, qualitative research

## Abstract

**Purpose:**

Anxiety and depression are important risk factors for dry eye disease (DED). The aims of this research are to identify the cause of anxiety and depression in DED patients and explore their strategies in coping with DED.

**Methods:**

This is a qualitative study based on semi-structured interviews, and the interviews records were analyzed through inductive thematic analysis. Participants were recruited from a large university affiliated hospital in the north of China, including 47 participants affected by depression and anxiety.

**Results:**

Analysis revealed the causes of anxiety and depression in DED patients could be divided into three major themes and nine subthemes: (1) From hospital: including difficulties in diagnosing and seeking medical advice, neglect or lack of attention from clinicians, low treatment satisfaction and complex comorbidities; (2) From daily life: including life satisfaction and well-being, changes in lifestyle pattern and changes in workstyle pattern; (3) From society: including burden of disease and reduction of social interaction. Most DED Patients with anxiety and depression were more likely to face the condition as well as receive treatments negatively, while the others tended to seek treatments unduly and blindly.

**Conclusion:**

This investigation offers new insights into the understanding difficulties in DED patients with anxiety and depression, and provides valuable guidance for supporting them to reduce depression and anxiety as well as improve prognosis.

## Introduction

Dry eye disease (DED) is a complex chronic, progressive ocular surface disorder, which is considered to be one of the most common eye diseases in modern society (1). Current studies indicate that the overall prevalence of DED worldwide ranges from 5 to 50%, and the prevalence is significantly higher in the Asian population compared to the Western population (2–6). The typical characteristic of DED is the homeostasis loss of tear film that is the important refractive and protective medium of the ocular surface. Therefore, such instability can further result in visual impairment, eye pain, ocular surface inflammation and even corneal perforation of DED patients, which seriously affect the quality of life and professional work of patients as well as bring a great burden to both medical security and socioeconomic systems (7–9).

Several research have identified the risk factors of DED including advanced age, female sex and smoking and anxiety (10, 11). Among them, anxiety has attracted widespread attention, as its special meaning in influencing the treatment and management of the chronic and progressive DED (1, 12). Many large retrospective studies, cohort studies and meta-analysis have proven the DED patients generally presented high level of depression and anxiety compared with healthy control (13–16). Although several interdisciplinary cooperation methods like referral to psychiatrists have been proposed, there is still a lack of efficient ways for ophthalmologic clinicians to cope and intervene with the anxiety and depression of DED patients (17).

Qualitative research has been proposed as an efficient way for understanding the psychological condition of patients including illness experiences and feelings, to optimize the treatment plan in the framework of “Narrative Evidence-Based Medicine” especially for chronic illness (18, 19). Several research have revealed the causes, coped strategies of low mood, and determined how to better intervene from the psychological perspective besides traditional treatment in various diseases including antenatal distress, inflammatory bowel disease, fibromyalgia, primary Sjögren’s Syndrome and primary open-angle glaucoma (20–24). However, there is still a lack of qualitative research focused on DED patients. In this study, we explored the experience of psychological distress in DED patients through interviews based on the qualitative approach, aiming to provide references for analyzing their anxiety and depression and to improve the management and treatment of DED patients in future.

## Materials and Methods

### Study Design and Patients

This is a descriptive qualitative study based on semi-structured interviews, and the standards of reporting meet with “The Standards for Reporting Qualitative Research” (SQRQ) guideline (25). DED Patients were recruited from January 2021 to June 2021 from Peking University Third Hospital and the participants inclusion criteria was followed as: (1) Meets diagnosis of DED according to TFOS DEWS II report (26), (2) Showing significant anxiety and depression, as well as Hamilton anxiety rating scale >14 or/and Hamilton depression rating scale >17, (3) Age >18, and demonstrate a positive attitude toward sharing their experience of illness. The participants who had other ocular conditions like glaucoma, and were unable to communicate properly including suffering from mental illness as well as speech impairment were excluded from this study to reduce the anchor and non-response bias. The sample size was identified by the principle of code saturation, and the recruitment was stopped if there were no additional new information or themes obtained after interviewing with three patients continuously (27, 28). The study was approved by Peking University Third Hospital Medical Science Research Ethics Committee, and all procedures were conducted under the guidance of Declaration of Helsinki. All participants signed informed consent before interviewing and approved their audio and transcript materials generated form the interview.

The face-to-face individual interviews with participants were performed by two researchers with both medical and epidemiological background (HY and WZ) in a quiet and private consultation room or online Tencent Meeting because of COVID-19. The main open-ended questions of semi-structured were “Has having dry eye changed your life?” “When do you experience anxiety or depression?” “Can you describe the cause of your anxiety or depression?” “Do you think you will be able to self-regulate the anxiety or depression in the future?” On this basis, the interviewer used more prompt statements to encourage patients for further sharing related opinions with minimal reservation (29). Each individual interviews lasted approximately 40 min, and were audio-recorded and then transcribed verbatim by the researchers.

### Data Analysis

The coding of transcript materials was conducted by NVivo software, and the determination of overarching themes and subthemes was based on inductive analysis rather than existing theoretical framework to expand the understanding of anxiety or depression in DED patients as much as possible (30, 31). The transcripts were de-identified and numbered according to the chronological order of the interview prior to coding, and the generally coding process were followed by: (1) reading transcripts repeatedly and screening meaning units, (2) defining themes, (3) indexing and classifying primary data based on the identified themes, and (4) reviewing the exist themes and update according to the new added materials. All the researchers had regular meetings to discuss and identify the key themes of current materials until no additional theme emerged, and the final decisions on controversial topics were made by senior doctors (YF).

## Results

A total of 47 patients were included in this study for semi-structured interviews, with the age of 44.9 ± 6.37 years. The majority were female (38/47), and the duration of disease was between 1 and 7 years (median: 5 years). The OSDI score, Hamilton anxiety rating scale and Hamilton depression rating scale are 35 ± 6, 18 ± 4, and 21 ± 3, respectively. The interviews of 47 participants were mainly concluded as following. Table 1 summarizes the themes and subthemes we determined according to the coding process about the cause of anxiety and depression in DED patients.

**TABLE 1:** Overview of thematic framework.

Themes	Subthemes
From hospital	Difficulties in diagnosing and seeking medical advice
	Neglect or lack of attention from clinicians
	Low treatment satisfaction
	Complex comorbidities
From daily life	Life satisfaction and well-being
	Changes in lifestyle pattern
	Changes in workstyle pattern
From social	Burden of disease
	Reduction of social interaction

### Analysis of Themes: From Hospital

#### Difficulties in Diagnosing and Seeking Medical Advice

For primary care, the diagnosis of DED was generally difficult due to the lack of relevant large equipment in assessing quality of the ocular surface. Relying solely on subjective examinations like Schirmer *I* test with high instability and limitations, which resulted in many mild cases not been diagnosed in time for early intervention and led to an exacerbation condition. When referred to a higher-level hospital, many doctors might also give different staging due to the complexity of DED classification, leaving patients with anxiety in following medical advice. However, not every patient could afford to see the doctor at a higher-level hospital due to the geographical distance or limited appointment of clinicians. In addition, the less severity with DED than blinding eye disease, at least for the visual acuity and visual fields, also made patients feel unworthy or pointless in expensive specialist outpatient of the higher-level hospital.

*“I started to feel dry and uncomfortable about 2 years ago and then I chose to go to the community health service where the doctor told me that your eyes were normal and there was nothing serious wrong with them, just a bit of visual fatigue and you needed to take a rest. Although I still found it a bit uncomfortable, I relaxed a bit when I heard that I was healthy. However, along with the symptoms getting worse later, the doctors in the community were still unable to provide effective diagnostic advice and they suggested me to go to a higher hospital. It was only then that I learned what dry eye was. I felt that I had lost the best time for treatment, which could lead to a poor prognosis for my DED” (Participant 1).*

*“I went to many hospitals and some doctors told me that my DED were due to poor function of the lid glands, but others told me it was due to bad lifestyle habits. They have given me different prescriptions and treatments, which one do you think I should trust and can you tell me the difference between them?” (Participant 22).*

*“I was 68 years old. I lived a long way from the hospital, and I could not walk alone to conduct regular follow-up visits without my children because of my pain joints and low physical-tolerance. However, they were busy with work, and I did not want to bother them if I just feel a little uncomfortable” (Participant 24).*

#### Neglect or Lack of Attention From Clinicians

During interviews, many patients complained that their doctors did not pay enough attention to DED, and even treated them indifferently, which amplified their anxiety and depression as a result of their illness. This lack of attention stemmed from the fact that many doctors believed that the disease was unimportant because of low probability in causing blindness and other serious complications.

*“The senior doctors may be busy with some complex eye diseases in clinic and DED seems to be a common and simple disease, so when I ask for more information or advices, they may feel impatient and even angry. I was afraid of such situation” (Participant 6).*

*“When I complained to my doctor about how hard I was feeling because of suffering DED, he always repeated the same thing saying that there was nothing wrong compared with other ocular diseases and even tried to refer me. I did not think he can empathize with my pain and maybe he does not want to spend time in treating me” (Participant 11).*

#### Low Treatment Satisfaction

Most patients have experienced anxiety and expression during the treatment of dry eye, and the common reason was the delayed improvement in symptoms after frequent medication. On this basis, many patients even sought health care everywhere and overmedicated as a result, which might exacerbate their symptoms and create a vicious cycle. In addition, several patients reported that their attending physician had told them that their signs have improved while the symptoms have not actually in terms of their feelings, which caused them to suspend the necessity and effectiveness of treatment.

*“Since I was diagnosed as DED, I had used the eyedrops, including artificial eyedrops and fluorometholone three times a day for several years, but still my ocular surface staining did not get significant improvement. I was so disappointed that I intended to quit up treatment but I was worried about if ceasing eyedrops, my symptoms would get more serious” (Participant 3).*

*“I had been to several hospitals for DED and each one seemed to prescribe a different medicine. The common thread was that I did not feel any benefit from the medication, although some doctors pointed that the symptoms of my ocular surface had improved. The disease did not seem that serious, but why was it so difficult to treat?” (Participant 8).*

#### Complex Comorbidities

In many cases, DED was also a secondary manifestation of many diseases like autoimmune diseases. Such patients generally presented the complex general condition and needed multiple treatments, therefore their anxiety and depression stemmed from many factors not just from DED.

*“I was diagnosed as primary Sjögren’s syndrome for 8 years and the initial symptom was DED. To date I had taken hydroxychloroquine (HCQ) for almost 8 years and various eyedrops for 9 years. Recently, I noticed that my vision had become blurred, and the right eye had distorted vision. I was so concerned that I went to ophthalmology clinic to do some examinations, and the doctor told me that I was diagnosed as macular edema and had better receive anti-VEGF therapy with drug-infusion in the vitreous cavity. The comorbidity might be attributed to the long-term use of HCQ, which scared me a lot and I was so worried about if I would lose my vision” (Participant 13).*

### Analysis of Themes: From Daily Life

#### Life Satisfaction and Well-Being

As a chronic condition affecting the sensory system, DED significantly reduced the life expectations in patients. Besides the uncomfortable feeling like dryness, hard to open eyes, frequent blinks, burning and grinding, the damage to the tear film might also affect their functional vision and many patients complained of themselves acting like those who were visually impaired. Especially for patients with moderate or severe DED, such discomfort could stay with them throughout the day, which made them get bored, anxious or depressed in everything they did. Such persistent negativity caused patients to feel incompetent as well as to lose hope and aspirations for their futural lives, and the life became utterly pointless to them.

*“During the daytime, my eyes felt like burning and needed to use eyedrops to keep the ocular surface wet. If I did not get eyedrops timely, I would feel my eyes like ant crawling in surface, which made me so angry and I kept rubbing my eyes. It was hard for me to devote full attention to do anything in this situation, which makes me feel so frustrated and depressed” (Participant 5).*

*“Every morning when I got up, my eyes felt really bad and I knew it means the bad day was starting again. I found it very difficult to get through such a bad day and I did not know how my life would be going on” (Participant 14).*

#### Changes in Lifestyle Pattern

Many patients had to change their lifestyle to accommodate the discomfort of DED, including altering travel patterns, recreational activities and diet structure. Although sometimes these changes were positive for health to a certain extent, patients still felt resistant because they believed the changes were forced by the disease instead of self-initiated option. Such senses of discomfort with their new lifestyle and loss of control over their lives triggered intense anxiety and depression.

*“I was not clear if the advanced age or DED made me feel so fatigue and my sleeping quality had been influenced a lot, including late night restlessness, early awakening and shorten sleeping duration. During the daytime, when I walked over 1 h, I had to stop for a rest, therefore I decreased many regular relaxation and activities. Besides, I was now eating a very light diet. It made me feel like I had lost a lot of joy in life, however, I could not change this predicament” (Participant 7).*

*“I could not endure activities that last for over 3 h, like reading or driving, especially at night when I felt the halos and lights are disorientating. Therefore, I must go home before the sun sets or calling for a driver. Most importantly, my doctors suggested me not to wear any contact lens, which was a huge challenge for a dress-up-loving girls” (Participant 30).*

#### Changes in Workstyle Pattern

For DED patients, the clinicians usually advised them to decrease the study and work time for avoiding prolonged screen time to prevent aggravation of DED symptoms. However, this recommendation was not feasible for many patients due to the nature of the profession like programmer, office clerk and student. The career advancement and physical health had become a dilemma for them, while for patients with severe dry eyes, they had no choice but to change the workstyle even jobs for relieving the symptoms of their eyes which sacrificed their development opportunities to some extent. In addition, many patients had to work with moisture chamber glasses, which attracted curious glances and inquisitive questions from colleagues.

*“I knew clearly that I should control the time of using eyes, however, I needed to study hard to prepare for college entrance examination. The enrollment pressure made me hard to stop my pace. When my eyes felt dry and uncomfortable, I became exhausted and very anxious” (Participant 4).*

*“I was a chef proficient in various cuisines and work in a busy restaurant. Since I suffered from DED, the smoke and working environment further deteriorated my ocular status. Although I used eyedrops frequently, my eye irritation including photophobia, sting and tearing could not be relieved totally. I had to decrease my working time and even consider that whether should I change another job?” (Participant 15).*

*“I had to wear* moisture chamber glasses *to keep my eyes comfortable when I worked on the computer, and even that I still could not keep up with the long hours. When I took eye drops frequently, my colleagues would ask me ‘Are you ok?,’ which made me a little uncomfortable and embarrassed. I had also put off a lot of overtime and had not completed my performance appraisal, I thought my boss might not promote me again” (Participant 21).*

### Analysis of Themes: From Social

#### Burden of Disease

Dry eye disease placed a huge socio-economic burden on society, families, and individuals, and the annual overall burden of DED on the hospitals in the United States was estimated at USD 3.84 billion as well as per patient at USD 771 and USD 1267 for patients with moderate and severe DED, respectively (32). Many of the drugs used for dry eyes like artificial tears, although covered by health insurance, were only available at limited monthly quota per patient in the context of controlling the medical expenditure, which might not fully meet the needs of patients with moderate and severe DED. In addition, several novel DED drugs such as cyclosporine A were not included in the China health insurance, while these drugs were usually more expensive. Such factor had brought a heavy economic burden to the both patients and their family who suffered from serious DED or needed long-term therapy, which further aggravated their negative emotions especially for patients who had not benefited from medication in the short term. Furthermore, the patients also worried about the potential damage to their economic and social rights like being fired due to the pattern shift in their daily life and work affected by DED.

*“I had to reduce the frequency of my DED appointments because my health insurance is inadequate (and conflicts with the cost of other illnesses), and my doctor said it was fine, however, I was still concerned that this may leave some changes in my condition unnoticed in time” (Participant 40).*

*“I thought the current medication for DED was too expensive, especially for someone like me who needs to use it frequently. My doctor had recommended some new medication to me before saying it was more suitable for my symptoms, however, it was too expensive and not covered by health insurance. And I did not feel significantly improvement of symptoms after using it for a period of time, so I asked my doctor to change the treatment plan” (Participant 2).*

*“Why are dry eye medications covered by health insurance often out of stock in some hospitals? Not being able to buy medication when my condition was severe was really distressing” (Participant 28).*

*“I suffered from DED due to the consistently working overtime. As a programmer, DED had seriously affected my productivity and I was afraid that my boss will fire me for this reason, after all, it was very difficult for me to find a new job in this situation” (Participant 31).*

#### Reduction of Social Interaction

Due to the widespread prevalence of electronic devices and the COVID-19 epidemic, social activities had shifted more to online, while it was very inconvenient for DED patients who cannot tolerate for long periods of screen viewing. In addition, DED also affected the accessibility of patients in current news through Apps, TV, and newspapers, which made them feel disconnected from society. Many patients felt lonely and depressed as a result and they further attributed these to DED, leading to negative feedback loop.

*“Before I had DED, my friends were in touch with me through WeChat. However, now I cannot return messages timely because I am not able to stare at the screen for long periods. Gradually, they stopped contacting with me and I felt it hard to fit in with them anymore” (Participant 41).*

*“I did not have the energy to care about the surrounding news and issues right now, I just wanted a quick escape from DED and got back to normal life” (Participant 19).*

*“It had been a long time since I last connected with my classmates and friends due to uncomfortable feelings in using phone and pad. They invited me to go out before, but I could not endure the stimulation of the wind or the sun to my eyes, so I turned them down. Now I felt like I only had my family by my side, however, once they got busy, I felt as if I had been abandoned by the world” (Participant 23).*

### Cope With Dry Eye Disease

Dry eye disease as well as anxiety and depression formed negative feedback that together affected the patient, which resulted in different options of patients in dealing with diseases. Chosen to constantly seeking for medical advice, leading to overtreatment while the opposite tended to cease the drug therapy and reject to transfer their lifestyle or workstyle. A small part of DED patients realized that they combined with anxiety or depression and its severity and started to ask for assistance like psychological medication. However, due to the chronic process and long-term treatment, the mental illness was also difficult for patients and doctors to cure totally in a short time, which indeed need great patience and perseverance.

*“In the hospitals, I met some patients whose symptoms were similar as mine, and through chatting with them, I found that their directions of therapies were different from mine. Some got better while some became worse, so I turned around multiple departments and attempted a variety of drugs according to the suggestion of different doctors. When my symptoms and feelings did not get significant improvement, I would trap in another types of anxiety” (Participant 32).*

*“Although I had used eyedrops for a period and acquired effective outcome to a certain extent, I would like to quit treatment as my symptoms were just mild and my work nature could not support long-term revisit. The advice of reducing the time of eye-using is almost impossible for me (a bank clerk) so it was not an appropriate choice and after enduring for a while, I may be likely to get relief. Consequently, the treatment seems a little meaningless” (Participant 39).*

*“After diagnosing with DED and using eyedrops for 5 months, I found myself really anxious about my effectiveness of therapy. If doctors told me that my signs were similar as the last time, I would feel like that the treatment was not beneficial for my diseases and was so disappointed and kept inquiring how could I get better. Realizing my mental abnormality, I also sought for psychologists for help and received psychomodulation therapy. The situation did not totally get control. Though I prepared for this long-term struggle with the encouragement of families and friends, it still was such a huge obstacle for me to conquer” (Participant 27).*

## Discussion

The relationship between DED and anxiety and depression has been clearly established in several cross-sectional studies as well as retrospective studies, and the previous study mainly assesses the negative emotion of patients and its impact on quality of life based on scales while there are few research focused on the qualitative method in exploring the broad range of their psychological states like anxiety and depression to our knowledge (33–35). This study analyzed the main causes of anxiety and depression and their coping strategies in 47 dry eye patients based on semi-structured interviews and qualitative analysis and the causes were further concentrated on three dimensions: from hospital, from daily life and from social, which aimed to provide better interventions or management of their treatment.

Hospital-related depression and anxiety were prominent themes articulated by DED patients in our research, which mainly could be attributed to the inconsistency of signs and symptoms in DED. Several studies have also reported similar results, Bartlett et al. (36) found the signs with symptoms in DED were heterogeneous, which might influence the correct assessment of the condition of patients. Kyei et al. (37) further reported only the blink rate and contrast sensitivity demonstrated significant correlation with OSDI among a wide range of the ocular surface examinations. Such property of DED might cause a major misunderstanding between patients and doctors if fail to communicate adequately, which would further induce conflicts, and aggravate the anxiety and depression of patients. Therefore, the relevant specialists and nurses should be encouraged to participate in trainings of expressing their empathy to DED patients to promote the positive interaction between them and patients, especially paying greater attention to their subjective symptoms.

Currently, China has established three-tier medical system, and continues to deepen the medical reform to implement the tiered health-care delivery system nationwide (38). Although first-level hospitals can cover the vast majority of urban and rural areas in China, their main functions are to provide basic medical services such as prevention, routine diagnoses, treatment and health care, they are usually lack of accurate examination equipment as well as experienced clinicians, which makes it difficult to make a clear diagnosis of DED and further results in delaying treatment and worse prognosis (39, 40). Therefore, it is important to raise awareness of DED among general practitioners and to develop novel portable ocular surface diagnostic instruments, while the former can be achieved by continuing education easily (41, 42). In addition, increasing economic investment of DED, including reducing the financial burden of patients through insurance and improving facilities of hospitals is also a promising way to improve its prognosis.

Dry eye disease significantly affects the vision of patients, inevitably limiting their ability to receive information and reducing their sense of participation in social and daily life especially with the prevalence of electronic social media. In addition to professional counseling for mental health, patient-to-patient communication should be further encouraged to alleviate their social anxiety like establishing patient support groups. It has been proven that the patient-to-patient communication was more effective than doctor-to-patient communication, which could improve patient compliance and confidence in terms of disorders of the sense organs, especially for patients with higher levels of education (43). In addition, DED could be categorized as the contested illnesses owing to its inconsistent symptoms and signs, and therefore the clinicians or social volunteers with adequate medical knowledge should deeply involve in such program to prevent deepening conflicts between doctor and the patient and amplifying the negative feelings of patients, which caused by communicating with the incorrect treatment information (44–46).

This study highlights the necessity of psychological intervention for DED patients who experience severe anxiety and depression. Many patients had already demonstrated as low compliance, doubt, resistance and self-abandonment to therapy under these negative emotions, which might further promote the progression of DED, and create a vicious cycle. In this context, systematic treatment of DED should be promoted, Yeo and Tong (47) also reported the DED patients demonstrated greater interest and acceptance of holistic treatments such as distracting their attention or sports compared with the single medication in a qualitative research about how to cope with DED. Except for conventional psychotherapy, several interventions have been proposed earlier for improving the subjective happiness, decreasing anxiety and depression score of DED patients including steam warming eye mask and cognitive behavior therapy (48, 49). More psychological interventions from a clinical perspective for DED patients need to be raised and evaluated in future based on the cause of anxiety and depression identified in this study such as establishing patient-support-group and other personalized communication program and treatment plan.

There are limitations in our study. This is a single-center study which might affect the generalizability of our findings despite the themes had saturation. However, the hospital where the study was conducted accepted numerous referrals requests each year from other areas, indicating the results was relatively robust at a national level. The screening of patients enrolled in this study was determined by Hamilton scales, which might overlook anxious or depressed DED patients who did not meet the clinical criteria. In addition, it was difficult to precisely distinguish whether the anxiety and depression stem from the personality of DED patients or from the illness limited to the cross-sectional nature of this study. Furthermore, there also were inner limitations of qualitative research including sample selection bias that only the patients who volunteered to share were included in the study.

## Data Availability Statement

The original contributions presented in the study are included in the article/supplementary material, further inquiries can be directed to the corresponding author.

## Ethics Statement

The studies involving human participants were reviewed and approved by the Peking University Third Hospital Medical Ethics Committee. The patients/participants provided their written informed consent to participate in this study.

## Author Contributions

HY and YF conceived and designed the entire study. HY and WZ conducted the main interviews with patients and wrote the initial draft of the manuscript. All authors were involved in the analysis of the code and made a substantial contribution to the article and approved the final manuscript.

## Conflict of Interest

The authors declare that the research was conducted in the absence of any commercial or financial relationships that could be construed as a potential conflict of interest.

## Publisher’s Note

All claims expressed in this article are solely those of the authors and do not necessarily represent those of their affiliated organizations, or those of the publisher, the editors and the reviewers. Any product that may be evaluated in this article, or claim that may be made by its manufacturer, is not guaranteed or endorsed by the publisher.
